# Association between pulse pressure levels and mortality in patients with septic shock: a retrospective cohort study

**DOI:** 10.3389/fmed.2026.1770215

**Published:** 2026-04-22

**Authors:** Yu Ji, Lisha Huang, Chang Cao, Wenyan Xiao, Tianfeng Hua, Min Yang

**Affiliations:** 1 Department of Critical Care Medicine The Second, The Second Affiliated Hospital of Anhui Medical University, Anhui, Hefei, China; 2 The Laboratory of Cardiopulmonary Resuscitation and Critical Care Medicine, The Second Affiliated Hospital of Anhui Medical University, Anhui, Hefei, China

**Keywords:** 28-day mortality, hemodynamic management, prognosis, propensity score matching, pulse pressure, septic shock

## Abstract

**Objective:**

Research on the impact of Pulse Pressure (PP) levels on Septic Shock patient prognosis is relatively limited. This study aims to analyze data from septic shock patients to investigate the prognostic effects of maintaining different PP levels during the early resuscitation phase.

**Methods:**

Data were extracted from the MIMIC-IV 3.1 database and septic shock patients meeting inclusion and exclusion criteria were collected from the Second Department of Critical Care Medicine at the Second Affiliated Hospital of Anhui Medical University. Patient information was gathered, and the average PP over the first 24 h in the ICU (PP_24__*h*_) was calculated. The optimal PP cutoff points were determined using X-tile software, and patients were divided into three groups based on PP_24__*h*_. Propensity score matching (PSM) was applied to adjust for confounding factors, and results outcomes were compared across groups. Subgroup analyses explored variations in results among different populations, and multivariate logistic regression further assessed the relationship between PP and outcomes. Finally, local data were used for generalizability assessment of the primary findings on 28-day mortality.

**Results:**

PP_24h_ maintained within 40–70 mmHg exhibited significantly higher survival rates. Subgroup analysis suggested that age significantly influenced the PP-mortality relationship: among elderly patients (>65 years), PP_24h_ > 70 mmHg was associated with the highest mortality risk, whereas in younger patients (≤65 years), PP_24h_ < 40 mmHg posed the greatest risk. Univariate and multivariate logistic regression confirmed that PP_24h_ > 70 mmHg or < 40 mmHg were independent risk factors for 28-day mortality in septic shock patients.

**Conclusion:**

In our study, maintaining PP levels between 40 and 70 mmHg during early resuscitation was associated with significantly lower 28-day mortality in septic shock patients. Our exploratory findings suggest that hemodynamic management strategies might need to consider patient age. Future studies are needed to investigate whether targeting an upper PP limit in elderly patients or avoiding low PP in younger patients could improve outcomes.

## 1 Introduction

Sepsis is a condition of life-threatening organ dysfunction caused by a dysregulated host response to infection (1). And the type that still requires vasoactive drugs to maintain blood pressure after adequate fluid resuscitation is called septic shock (2, 3). Mortality from sepsis has remained high in recent years, with approximately 11 million deaths related to sepsis each year globally. In China, the in-hospital mortality rate for sepsis reaches 28.4% (4, 5). Hemodynamics support is one of the core aspects of septic shock treatment; however, clinical observations revealed that patients treated with a mean arterial pressure (MAP) ≥ 65 mmHg as a target according to guideline recommendations had an unsatisfactory prognosis, suggesting that a single MAP index may not be sufficient for a comprehensive assessment of circulatory status (6, 7). Pulse pressure (PP) is defined as the difference between systolic and diastolic blood pressure and reflects the energy output of the heart to the arterial system (8, 9). It has been shown by teams to correlate with cardiac output and vascular elasticity to better reflect the hemodynamics state. But in septic shock fewer studies have been done on PP (10, 11). This discrepancy highlights a critical knowledge gap: the relationship between PP and mortality is likely non-linear, yet most studies have focused on unidirectional thresholds without identifying an optimal “safety range” for the early resuscitation phase. To address this, our study analyzed high-granularity data from the MIMIC-IV database and an external clinical cohort. We aimed to reconcile these conflicting perspectives by determining whether an optimal PP range exists and investigating the independent prognostic association of deviations—both low and high—from this range on 28-day mortality.

## 2 Materials and methods

### 2.1 Clinical data

Source of research subjects: 1. MIMIC-IV (Medical Information Mart for Intensive Care). Our research team has formally obtained access to this database (authorization number: 60356492) for data download and analysis. 2. Local data: Another portion of the research data originated from local databases; specifically patient records of septic shock admitted to the Second Department of Critical Care Medicine at the Second Affiliated Hospital of Anhui Medical University from September 2021 to September 2024 (Ethics Approval Number: SL-YX2024-203).

### 2.2 Inclusion and exclusion criteria

Participants must satisfy the following conditions: Diagnosed sepsis with sequential organ failure score ≥ 2, dependence on vasoactive drugs to maintain a MAP ≥ 65 mmHg and a serum lactate concentration of > 2 mmol/L remained after fluid resuscitation therapy. Exclusion criteria: (1) age < 18 years; (2) women during pregnancy; (3) patients who received extracorporeal membrane oxygenation and aortic balloon counter pulsation; and (4) patients who were discharged or died within 24 h of admission to the ICU.

### 2.3 Observation indicators

X-tile is a bioinformatics tool developed by Yale University, specifically designed for biomarker evaluation and outcome-based critical point optimization, its core functionality employs the “enumeration method” to assist researchers in determining the optimal cutoff value for continuous independent variables within survival data, thereby enabling grouped analysis (12, 13). In the current study, we used the X-tile software to identify optimal cutoff points for grouping based on 24 h mean pulse pressure (PP24h), survival time, and the pre-specified primary endpoint of 28-day mortality and clinical outcomes. Invasive blood pressure changes, laboratory tests, outcomes, invasive operations, comorbidities, and condition scores were collected after ICU admission for patients who met the NAE criteria. Basic patient information included age, gender, length of ICU stays, and survival time. Blood pressure included invasive systolic blood pressure, invasive diastolic blood pressure, and mean arterial pressure, and PP was derived from invasive systolic blood pressure—invasive diastolic blood pressure, PP and MAP were calculated hourly and averaged over 24 h to obtain the mean 24 h mean arterial pressure (MAP24h) and PP24h. Laboratory tests included white blood cell count, hemoglobin, platelet count, γ- glutamylase, creatinine, blood potassium, and blood sodium. Where a variable is recorded multiple times, the worst value shall be selected based on existing clinical consensus and guidelines. Here, “worst” is defined as the pathophysiological state indicated by that variable which is most strongly associated with poorer clinical outcomes. Poorer clinical outcomes primarily include: higher risk of organ failure, longer ICU stay, or higher mortality rate. Invasive operations included the presence or absence of mechanical ventilation, CRRT. comorbidities included myocardial infarction, diabetes mellitus, hypertension, malignancy, chronic kidney disease and chronic obstructive pulmonary disease. Condition scores included SOFA score, Simplified Acute Physiological Function Scoring System.

### 2.4 Statistical methods

All statistical tests were two-sided with a pre-specified significance level of α = 0.05. Reported 95% confidence intervals (CIs) correspond to this α = 0.05 significance level. *P* < 0.05 was considered statistically significant only for the primary endpoint (28-day mortality) analysis. In this study, variables with a missing rate of more than 20% were excluded from the final study analysis. For variables with a missing rate of less than 5%, the mean value of the variable was used to fill in. For variables with a missing rate between 5 and 20%, linear interpolation was used to compensate for this. We used SPSS26 software to implement a series of data analysis steps covering data filling, 1:1 propensity score matching, and statistical analysis of baseline and outcome data. Propensity score matching in the study was set with a caliper value of 0.02 and a 1:1 matching ratio was used. For continuous data following a normal distribution, the mean and standard deviation ($\bar{x}$ ± s) are used for presentation, and a *t*-test is employed to compare two sets of data. For continuous data that were not normally distributed, we instead used median and interquartile spacing to describe them, and the Mann-Whitney U test to analyses the differences between the two sets of data. For categorical data, we used percentages for presentation and chi-square tests to assess differences between the two groups. In addition, we conducted univariate and multivariate logistic regression analyses using SPSS26 software to explore the effect of factors such as pulse pressure grouping on 28-day mortality in patients with septic shock. Finally, we performed Kaplan-Meier survival analyses and Log-rank tests with the help of R. We also carried out in-depth subgroup analyses and drew corresponding subgroup forest plots. The primary endpoint of this study was 28-day all-cause mortality, which was pre-specified for confirmatory statistical analysis. The 3- and 90-day mortality data reported in the results were exploratory analyses without pre-specified hypotheses, and their results were descriptive only. Only the primary endpoint (28-day mortality) was subjected to confirmatory statistical analysis without multiplicity adjustment. All other analyses, including 3-day/90-day mortality comparisons, subgroup analyses, and pairwise propensity score-matched comparisons, were exploratory in nature. No adjustment for multiple comparisons was performed for these exploratory analyses, and their *P*-values should be interpreted as descriptive rather than confirmatory.

## 3 Results

### 3.1 Study population and optimal cut-off determination

**FIGURE 1:**
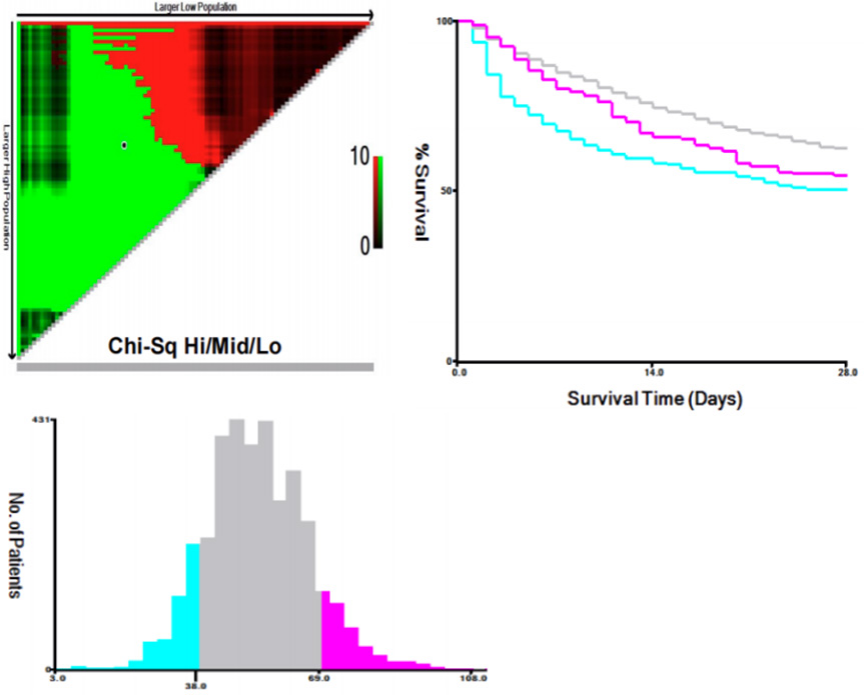
The optimal cutoff point for PP24h by X-tile.

A total of 3,668 patients with septic shock from the MIMIC-IV database (v3.1) met the inclusion criteria. Additionally, an external cohort of 316 patients was recruited from the Second Affiliated Hospital of Anhui Medical University. Using X-tile software to maximize the chi-square statistic for 28-day mortality, the optimal prognostic cut-off points for PP24h were identified as 38 mmHg and 69 mmHg (χ^2^max = 53.40, *P* < 0.001). For clinical practicality, these thresholds were rounded to 40 mmHg and 70 mmHg, respectively. This categorization remained statistically significant (χ^2^max = 47.68, *P* < 0.001) and was used to stratify patients into three groups: Group 1 (High PP, > 70 mmHg), Group 2 (Reference/Normal PP, 40–70 mmHg), and Group 3 (Low PP, < 40 mmHg) (Figure 1).

### 3.2 Baseline characteristics before and after

To account for baseline imbalances, we performed pairwise propensity score matching (1:1 ratio) for the abnormal PP groups against the reference group (Group 2) and against each other. Group 1 vs. Group 2 (High vs. Normal): Before matching, Group 1 (*n* = 330) and Group 2 (*n* = 2864) showed significant baseline differences. After matching, 325 pairs were generated. Covariates including age, gender, and SOFA scores were well-balanced (Standardized Mean Difference < 0.1), although the prevalence of hypertension remained slightly higher in Group 1 (*P* < 0.05), a residual imbalance acknowledged in our limitations. Group 3 vs. Group 2 (Low vs. Normal): Before matching, Group 3 (*n* = 474) differed significantly from Group 2. Post-matching (*n* = 467 pairs), all baseline variables, including lactate levels and comorbidity profiles, were comparable between groups. Group 3 vs. Group 1 (Low vs. High): A direct comparison yielded 159 matched pairs. Overall, PSM effectively reduced baseline heterogeneity, providing a more comparable set of cohorts for outcome analysis (Tables 1–3).

**TABLE 1:** Baseline condition before and after propensity score matching in the group one.

Variant	Subgroups (pre-PSM)		*P*	Subgroups (post PSM)		*P*
	G2:40–70 mmHg (2864)	G1: > 70 mmHg (330)		G2’: 40–70 mmHg (325)	G1’: > 70 mmHg (325)	
Female	1095(38%)	161(49%)	< 0.001	153(47%)	157(48%)	0.753
≤65 year	1268(44%)	76(23%)	< 0.001	92(28%)	75(23%)	0.127
>65 year	1596(56%)	254(77%)		233(72%)	250(77%)	
High blood pressure	1246(44%)	148(45%)	0.641	118(36%)	148(46%)	0.017
Diabetes	900(31%)	170(52%)	< 0.001	161(50%)	166(51%)	0.695
Myocardial infarction	215(8%)	43(13%)	0.073	31(10%)	34(10%)	0.695
Chronic kidney disease	480(17%)	96(29%)	< 0.001	104(32%)	93(29%)	0.348
Malignant tumor	411(14%)	67(20%)	0.004	60(18%)	66(20%)	0.552
Chronic obstructive pulmonary disease	172(6%)	27(8%)	0.121	30(9%)	26(8%)	0.576
Lactic acid (mmol/L)	3.2(2.5–4.6)	3.2(2.5–4.5)	0.511	3.2(2.5–4.7)	3.2(2.5–4.4)	0.802
White blood cell count ( × 10^9^/L)	13.4(9.2–18.9)	13.1(9–18)	0.657	13.8(9.8–19.9)	13.1(9.3–18)	0.377
Hemoglobin (g/dL)	10(8.4–11.6)	9.6(8.1–11.6)	0.129	9.8(8.4–11.4)	9.7(8.1–11.6)	0.81
Blood platelet ( × 10^9^/L)	154(110–216)	158(16–228)	0.161	170(121–239)	158(116–228)	0.195
Serum Potassium(mmol/L)	4.2(3.8–4.7)	4.3(3.9–4.8)	0.044	4.4(3.9–4.8)	4.3(3.9–4.8)	0.482
Serum Sodium (mmol/L)	139(136–142)	139(136–141)	0.419	139(135–142)	139(137–141)	0.448
Alanine Aminotransferase(U/L)	31(18–77)	31(17–78)	0.959	28(17–73)	32(17–78)	0.413
Creatinine (mg/dL)	1.1(0.8–1.7)	1.2(0.9–1.9)	< 0.001	1.2(0.8–2)	1.2(0.9–1.9)	0.578
SOFA	8(6–11)	8(5–10)	0.02	8(5–11)	8(5–10)	0.136
Sepsis II	46(35–57)	47(39–57)	0.052	48(38–60)	47(39–57)	0.556
MAP_24h_(mmHg)	74(70–78)	75(71–82)	< 0.001	75(71–81)	75(71–81)	0.962

**TABLE 2:** Baseline condition before and after propensity score matching in the group two.

Variant	Subgroups (pre-PSM)		*P*	Subgroups (post PSM)		*P*
	G2: 40–70 mmHg (2864)	G3 : < 40 mmHg (474)		G2^”^: 40–70 mmHg (467)	G3^”^: < 40 mmHg (467)	
Female	1095(38%)	173(36%)	0.471	186(40%)	173(37%)	0.382
≤65 year	1268(44%)	308(65%)	< 0.001	298(64%)	301(64%)	0.838
>65 year	1596(56%)	166(35%)		169(36%)	166(36%)	
High blood pressure	1246(44%)	156(33%)	< 0.001	143(31%)	156(33%)	0.362
Diabetes	900(31%)	98(21%)	< 0.001	101(22%)	98(21%)	0.811
Myocardial infarction	215(8%)	49(10%)	0.034	42(9%)	48(10%)	0.506
Chronic kidney disease	480(17%)	70(15%)	0.279	81(17%)	70(15%)	0.328
Malignant tumor	411(14%)	55(12%)	0.11	51(11%)	54(12%)	0.756
Chronic obstructive pulmonary disease	172(6%)	29(6%)	0.924	30(6%)	28(6%)	0.786
Lactic acid (mmol/L)	3.2(2.5-4.6)	3.8(2.6–6)	< 0.001	3.5(2.6–5.35)	3.7(2.6–5.95)	0.254
White blood cell count (×10^9^/L)	13.4(9.2–18.9)	14.3(8.6–20.6)	0.502	14(9.1–19.85)	14.3(8.6–20.6)	0.784
Hemoglobin (g/dL)	10(8.4–11.6)	11.4(9.6–13.3)	< 0.001	11(9.2–13.2)	11.4(9.6–13.3)	0.129
Blood platelet ( × 10^9^/L)	154(110–216)	193(124–272)	< 0.001	182(120–259)	192(123–272)	0.244
Serum potassium (mmol/L)	4.2(3.8–4.7)	4.4(3.8–5.1)	< 0.001	4.3(3.8–4.9)	4.4(3.8–5)	0.708
Serum sodium (mmol/L)	139(136–142)	138(135–141)	0.159	139(135–142)	138(135–141)	0.754
Alanine Aminotransferase (U/L)	31(18–77)	46(22–154)	< 0.001	37(21–122.5)	45(22–142)	0.138
Creatinine (mg/dL)	1.1(0.8–1.7)	1.3(0.9–2.1)	< 0.001	1.2(0.9–2.1)	1.3(0.9–2.05)	0.573
SOFA	8(6–11)	10(7–12)	< 0.001	10(7–13)	10(7–12)	0.962
Sapsi II	46(35–57)	50(40–62)	< 0.001	50(38–63)	49(40–61)	0.572
MAP_24h_ (mmHg)	74(70–78)	75(70–79)	0.258	74(70–79)	75(70–79)	0.302

**TABLE 3:** Baseline condition before and after propensity score matching in the group three.

Variant	Subgroups (pre-PSM)		*P*	Subgroups (post PSM)		*P*
	G3: > 70 mmHg (474)	G1: < 40 mmHg (330)		G3”’ > 70 mmHg (159)	G1”’: < 40 mmHg (159)	
Female	173(36%)	161(49%)	0.001	78(49%)	71(45%)	0.431
≤65 year	308(65%)	76(23%)	< 0.001	60(38%)	71(45%)	0.21
>65 year	166(35%)	254(77%)		99(62%)	88(55%)	
High blood pressure	156(33%)	148(45%)	0.001	64(40%)	66(42%)	0.82
Diabetes	98(21%)	170(52%)	0.001	57(36%)	53(33%)	0.637
Myocardial infarction	49(10%)	43(13%)	0.987	19(12%)	18(11%)	0.861
Chronic kidney disease	70(15%)	96(29%)	< 0.001	43(27%)	40(25%)	0.702
Malignant tumor	55(12%)	67(20%)	0.001	28(18%)	21(130%)	0.277
Chronic obstructive pulmonary disease	29(6%)	27(8%)	0.258	17(11%)	12(8%)	0.33
Lactic acid (mmol/L)	3.8(2.6–6)	3.2(2.5–4.5)	< 0.001	3.3(2.55–4.6)	3.5(2.5–5.05)	0.7
White blood cell count (×109/L)	14.3(8.6–20.6)	13.1(9–18)	0.393	13.1(8.9–18.35)	13.9(8.05–20.75)	0.781
Hemoglobin (g/dL)	11.4(9.6–13.3)	9.6(8.1–11.6)	< 0.001	10.6(8.8–12.4)	10.6(8.8–12.1)	0.446
Blood platelet (×109/L)	193(124–272)	158(16–228)	0.002	168(119–247)	167(113–232)	0.88
Serum potassium (mmol/L)	4.4(3.8–5.1)	4.3(3.9–4.8)	0.222	4.4(3.9–5)	4.3(3.7–4.9)	0.238
Serum sodium (mmol/L)	138(135–141)	139(136–141)	0.103	138(135–142)	139(136–141)	0.558
Alanine aminotransferase (U/L)	46(22–154)	31(17–78)	< 0.001	37(20–70)	42(19–132)	0.869
Creatinine (mg/dL)	1.3(0.9–2.1)	1.2(0.9–1.9)	0.271	1.3(1–1.95)	1.2(0.9–2.05)	0.276
SOFA	10(7–12)	8(5–10)	< 0.001	9(6–11)	8(6-11)	0.925
Sepsi II	50(40–62)	47(39–57)	0.011	49(40–63)	47(39–59)	0.321
MAP24h (mmHg)	75(71–82)	75(70–79)	0.01	75(71–80)	77(72–81)	0.249

### 3.3 Primary and secondary outcomes in the matched cohorts

28-day mortality was the pre-specified primary endpoint of this study. The 3-day and 90-day mortality data presented below were exploratory findings from *post-hoc* analyses, not pre-specified study endpoints. High PP vs. Normal PP (Group 1 vs. Group 2): In the matched cohort (*n* = 325/group), patients with High PP (>70 mmHg) exhibited a significantly higher 28-day mortality compared to those with Normal PP (34% vs. 26%, *P* = 0.027). The rates of 3-day mortality and 90-day mortality, which were exploratory outcomes, did not differ significantly between these matched groups. No significant differences were observed in the utilization of organ support (mechanical ventilation, CRRT) or ICU length of stay (Table 4). Low PP vs. Normal PP (Group 3 vs. Group 2): In the matched cohort (*n* = 467/group), Low PP ( < 40 mmHg) was associated with significantly worse outcomes compared to Normal PP. Specifically, the Low PP group had higher 28-day mortality (42% vs. 32%, *P* = 0.001) and 3-day mortality (14% vs. 9%, *P* = 0.041). However, 90-day mortality, an exploratory outcome, did not reach statistical significance in the matched analysis (Table 5). Low PP vs. High PP (Group 3 vs. Group 1): In the matched comparison between the two extreme groups (*n* = 159/group), patients with High PP (>70 mmHg) had a relatively lower 3-day mortality compared to the Low PP group (5% vs. 12%, *P* = 0.048), This exploratory descriptive finding suggests that low pulse pressure may be associated with more immediate early-phase lethality (Table 6).

**TABLE 4:** Outcome indicators of the group one before and after propensity score matching.

Variant	Subgroups (pre-PSM)		*P*	Subgroups (post PSM)		*P*
	G2: 40–70 mmHg (2864)	G1: > 70 mmHg (330)		G2’: 40–70 mmHg (325)	G1’: > 70 mmHg (325)	
ICU days of hospitalization	11(6,19)	11(5,18)	0.249	12(7,21)	11(5,18)	0.915
Whether CRRT is performed	448(16%)	70(21%)	0.09	54(17%)	70(22%)	0.11
Mechanical ventilation or not	2445(85%)	274(83%)	0.258	280(86%)	270(83%)	0.277
90d mortality rate	1258(44%)	178(54%)	0.001	163(50%)	175(54%)	0.346
28d mortality rate	753(26%)	115(35%)	0.001	86(26%)	112(34%)	0.027
3d mortality rate	151(5%)	14(4%)	0.423	20(6%)	13(4%)	0.211

**TABLE 5:** Outcome indicators of the group two before and after propensity score matching.

Variant	Subgroups (pre-PSM)		*P*	Subgroups (post PSM)		*P*
	G2: 40–70 mmHg (2,864)	G3 : < 40 mmHg (474)		G2”: 40–70 mmHg (467)	G3”: < 40 mmHg (467)	
ICU days of hospitalization	11(6.29)	8(5.18)	0.001	10(5.20)	9(5.19)	0.909
Whether CRRT is performed	448(16%)	124(26%)	< 0.001	113(24%)	119(25%)	0.65
Mechanical ventilation or not	2445(85%)	370(78%)	< 0.001	399(84%)	364(77%)	0.003
90d mortality rate	1258(44%)	263(55%)	< 0.001	238(51%)	257(55%)	0.213
28d mortality rate	753(26%)	204(43%)	< 0.001	152(32%)	199(42%)	0.001
3d mortality rate	151(5%)	67(14%)	< 0.001	44(9%)	64(14%)	0.041

**TABLE 6:** Outcome indicators of the group three before and after propensity score matching.

Variant	Subgroups (pre-PSM)		*P*	Subgroups (post PSM)		*p*
	G1: < 40 mmHg (330 cases)	G3 > 70 mmHg (474 cases)		G1^”’^: < 40 mmHg (159 cases)	G3^”’^> 70 mmHg (159 cases)	
ICU days of hospitalization (days)	11(5.18)	8(5.18)	0.15	9(6.18)	7(5.12)	0.816
Whether CRRT is performed	70(21%)	124(26%)	0.107	44(28%)	31(19%)	0.086
Mechanical ventilation or not	274(83%)	370(78%)	0.082	129(81%)	114(72%)	0.048
90d mortality rate	178(54%)	263(55%)	0.665	92(58%)	82(52%)	0.260
28d mortality rate	115(35%)	204(43%)	0.002	68(43%)	65(41%)	0.733
3d mortality rate	14(4%)	67(14%)	< 0.001	8(5%)	19(12%)	0.048

### 3.4 Survival analysis

**FIGURE 2:**
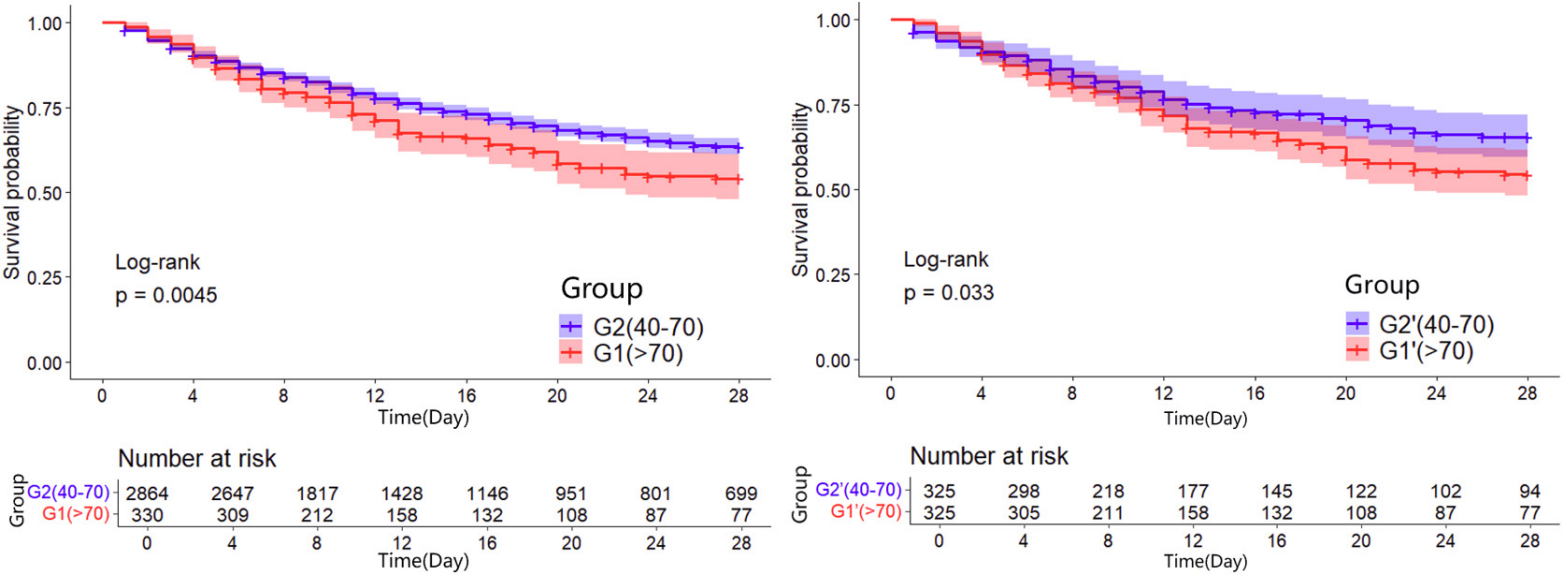
28d Kaplan-Meier survival curves of patients in the group one before and after propensity matching.

**FIGURE 3:**
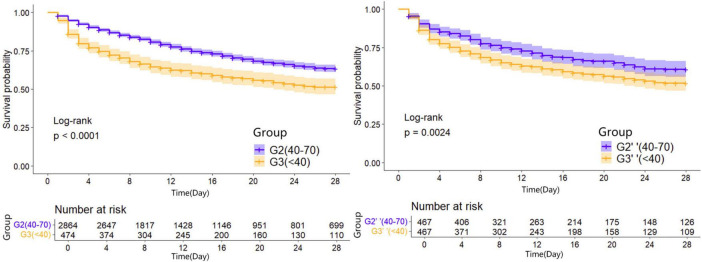
28d Kaplan-Meier survival curves of patients in the group two before and after propensity matching.

**FIGURE 4:**
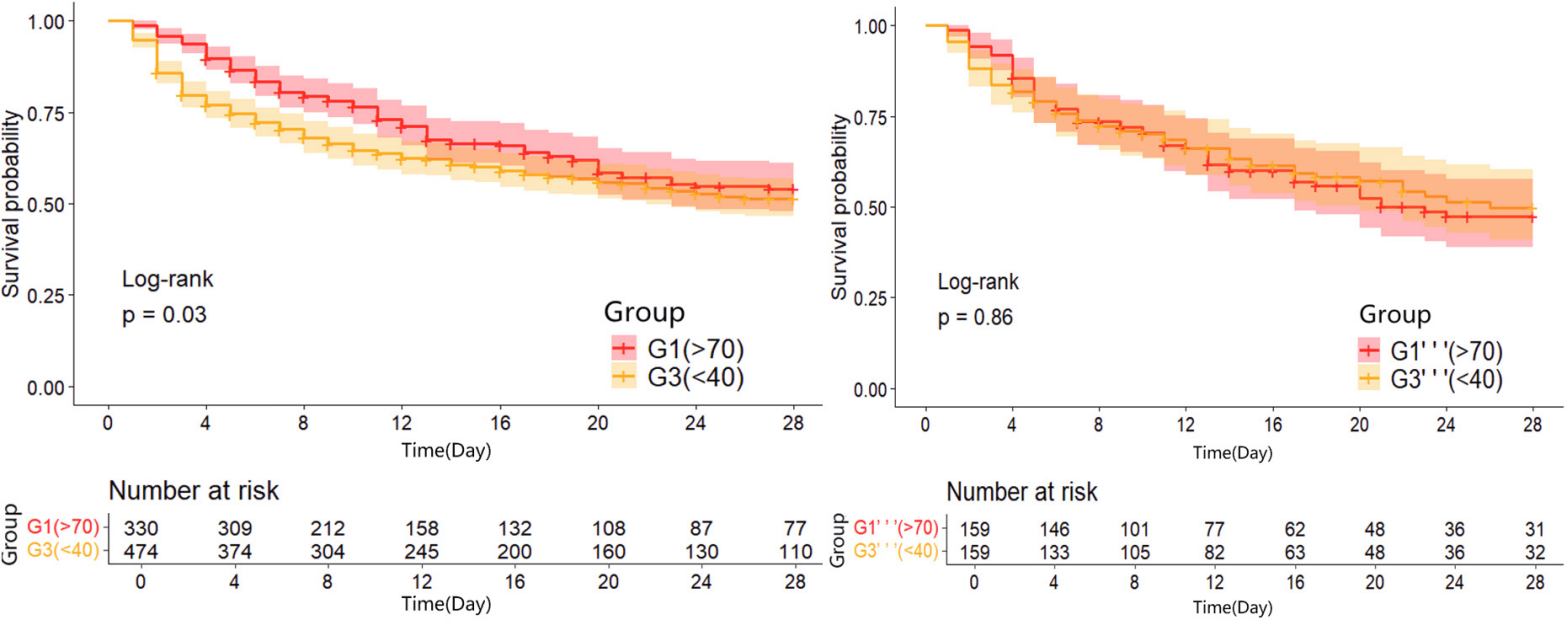
28d Kaplan-Meier survival curves of patients in the group three before and after propensity matching.

Kaplan-Meier survival curves were generated to visualize the time-to-event data. In the unadjusted analysis, patients maintaining PP24h within the 40–70 mmHg range demonstrated the highest survival probability. Consistent with this, in the propensity-matched cohorts, the Normal PP group (40–70 mmHg) continued to show a survival advantage over both the High PP group ( > 70 mmHg) and the Low PP group (<40 mmHg) (Log-rank *P* < 0.05 for both comparisons). The survival trajectories of the two abnormal groups (Low and High PP) diverged from the reference group but did not differ significantly from each other in the long term (Figures 2–4).

### 3.5 Exploration in an external cohort (generalizability analysis)

**FIGURE 5:**
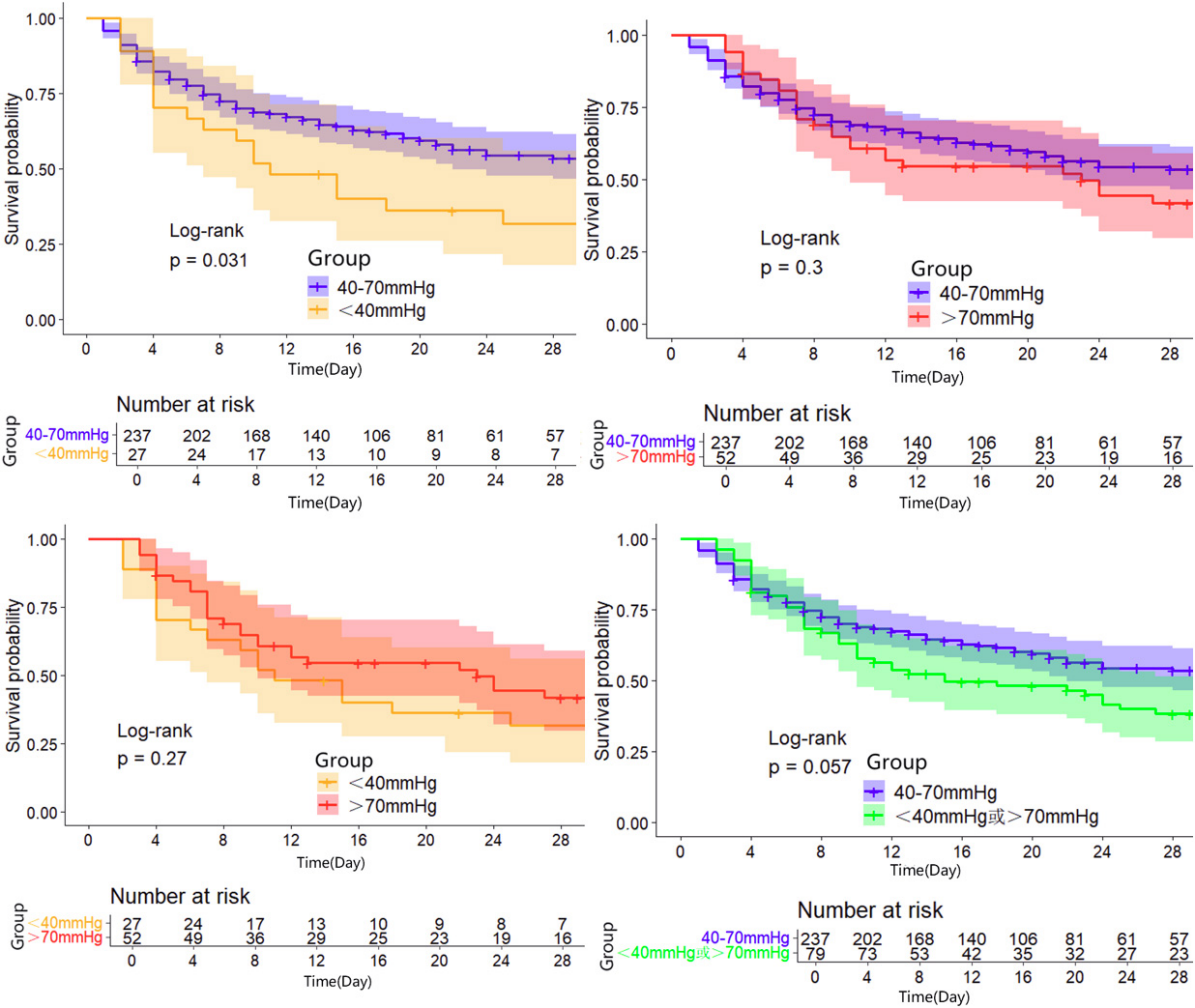
28d survival curves of patients in the local database.

To assess the generalizability of our findings, we analyzed an external cohort of 316 septic shock patients from the Second Affiliated Hospital of Anhui Medical University (2021–2024). Consistent with the MIMIC-IV derivation cohort, patients falling outside the 40–70 mmHg PP range exhibited poorer clinical outcomes (Table 7). Low PP ( < 40 mmHg): Patients in this group (*n* = 27) had significantly higher rates of CRRT use (56% vs. 27%, *P* = 0.002), mechanical ventilation (81% vs. 60%, *P* = 0.029), and 28-day mortality (67% vs. 41%, *P* = 0.009) compared to the Normal PP group (*n* = 237). High PP ( > 70 mmHg): Patients in this group (*n* = 52) also showed elevated 28-day mortality (58% vs. 41%, *P* = 0.006) and 90-day mortality (63% vs. 42%, *P* = 0.005), an exploratory outcome, although organ support requirements were not significantly increased. Kaplan-Meier analysis of this external cohort (Figure 5) mirrored the primary results, showing that patients within the 40–70 mmHg range had the most favorable survival probability (*P* = 0.031 compared to the Low PP group).

**TABLE 7:** Outcome indicators of the local database.

Variant	40∼70 mmHg (237 cases)	Another pp ( > 70 mmHg or < 40 mmHg) (79 cases)	P*	> 70 mmHg (52 cases)	P*	< 40 mmHg (27 cases)	P*
ICU days of hospitalization (days)	7(3.12)	8(4.17)	0.024	8(4.17)	0.024	8(4.15)	0.163
Whether CRRT is performed	63(27%)	30(38%)	0.054	15(29%)	0.739	15(56%)	0.002
Mechanical ventilation or not	142(60%)	53(67%)	0.256	31(60%)	0.256	22(81%)	0.029
90d mortality rat	100(42%)	55(70%)	< 0.001	33(63%)	0.005	22(81%)	< 0.001
28d mortality rate	96(41%)	46(58%)	0.006	28(54%)	0.078	18(67%)	0.009
3d mortality rate	21(9%)	3(4%)	0.143	0	0.026	3(11%)	0.7

P*Comparison of the values obtained with patients whose pulse pressure was 40–70 mmHg.

### 3.6 Subgroup and interaction analyses

**FIGURE 6:**
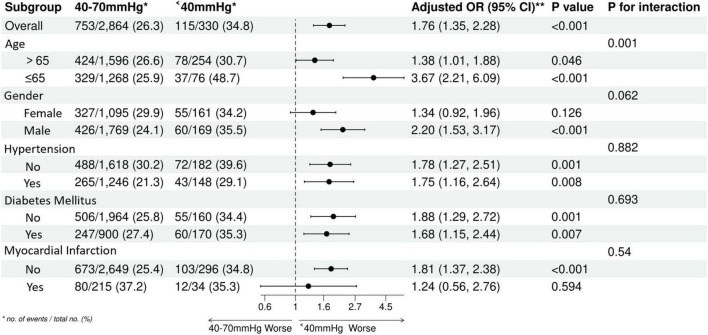
Subgroup analysis forest plot in the group one.

**FIGURE 7:**
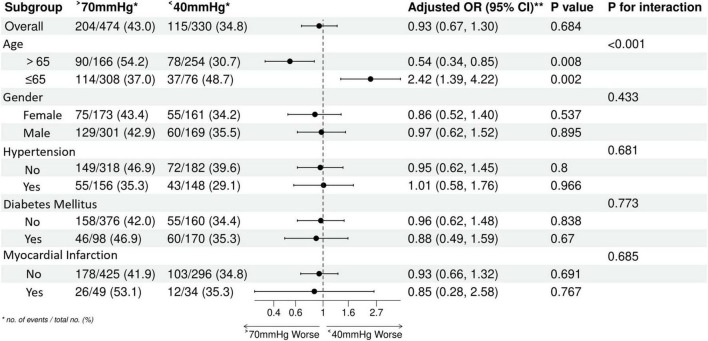
Subgroup analysis forest plot in the group two.

**FIGURE 8:**
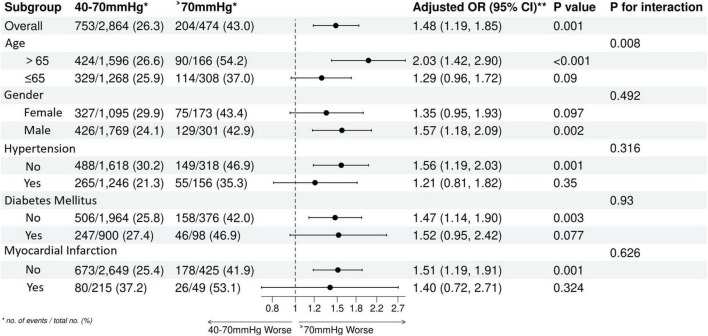
Subgroup analysis forest plot in the group three.

This was an exploratory subgroup analysis without pre-specified hypotheses and no adjustment for multiple comparisons. The results should be interpreted with caution. We utilized Forest plots to explore potential effect modifiers on the relationship between Pulse Pressure and mortality (Figures 6–8). The generally unfavorable association of abnormal PP (either High or Low) with mortality was consistent across most subgroups, including gender, diabetes, and hypertension status. Notably, an interaction with Age was observed (P for interaction < 0.05). In elderly patients (>65 years), a High PP (>70 mmHg) was more strongly associated with increased mortality risk compared to younger patients. Conversely, the detrimental association of Low PP (<40 mmHg) was prominent across age groups but showed varying magnitudes. This suggests that age may modulate the prognostic impact of pulse pressure, potentially due to age-related changes in vascular compliance.

### 3.7 Multivariable analysis of prognostic indicators

To evaluate the independent association between PP categories and 28-day mortality, we performed multivariable logistic regression. First, univariate analysis screened for potential predictors (Table 8). Variables with *P* < 0.05, including age, lactate, hemoglobin, and comorbidities, were entered into the multivariable model (Table 9). After adjusting for these confounders, both High PP24h (>70 mmHg) and Low PP24h (<40 mmHg) remained independently associated with increased 28-day mortality compared to the reference range. Other significant indicators included elevated lactate levels and hemoglobin. The multivariable model demonstrated fair discrimination with a C-index of 0.752. These results support the hypothesis that deviations in pulse pressure—both upward and downward—are independent markers of poor prognosis in septic shock.

**TABLE 8:** Univariate logistic regression analysis was conducted to explore the effects of different variables on the 28d mortality of patients with septic shock.

Variant	Regression coefficient	Standard error	Wald statistic	*P*	OR	95% CI	
						Lower limit	Limit
> 65 year	0.015	0.073	0.042	0.838	1.015	0.88	1.171
Male	–0.216	0.074	8.575	0.003	0.805	0.697	0.931
High blood pressure	–0.498	0.076	43.377	<0.001	0.608	0.524	0.705
Diabetes	0.07	0.078	0.823	0.364	1.073	0.922	1.249
Myocardial infarction	0.507	0.124	16.588	<0.001	1.66	1.301	2.119
Chronic kidney disease	0.153	0.094	2.685	0.101	1.166	0.97	1.4
Chronic obstructive pulmonary disease	0.47	0.141	11.138	0.001	1.6	1.214	2.109
Malignant tumor	0.199	0.1	3.915	0.048	1.22	1.002	1.485
PP_24h_ > 70 mmHg	0.405	0.123	10.836	0.001	1.5	1.178	1.909
PP_24h_ < 40 mmHg	0.751	0.102	54.127	<0.001	2.118	1.734	2.587
White blood cell count ( × 10^9^/L)	0.018	0.004	22.721	<0.001	1.018	1.011	1.026
Hemoglobin (g/dL)	0.083	0.015	31.306	<0.001	1.086	1.055	1.118
Blood platelet ( × 10^9^/L)	0.001	0	12.526	<0.001	1.001	1.001	1.002
Alanine aminotransferase (U/L)	0	0	5.285	0.022	1	1	1
Creatinine (mg/dL)	0.232	0.025	83.676	<0.001	1.261	1.2	1.326
Serum potassium (mmol/L)	0.154	0.043	12.566	<0.001	1.167	1.071	1.27
Serum sodium (mmol/L)	–0.027	0.007	15.168	<0.001	0.974	0.961	0.987
Lactic acid (mmol/L)	0.17	0.014	143.057	<0.001	1.186	1.153	1.219
SOFA	0.172	0.01	288.215	<0.001	1.188	1.164	1.212
SAPS II	0.046	0.003	317.612	<0.001	1.048	1.042	1.053
MAP_24h_ (mmHg)	0.002	0.005	0.24	0.625	1.002	0.993	1.012

**TABLE 9:** Multivariate logistic regression analysis to explore factors influencing 28d mortality in patients with septic shock.

Variant	Regression coefficient	Standard error	Wald statistic	*P*	OR	95% CI	
						Lower limit	Limit
Male	–0.282	0.084	11.319	0.001	0.755	0.64	0.889
High blood pressure	–0.351	0.084	17.3	< 0.001	0.704	0.597	0.831
Myocardial infarction	0.324	0.153	4.45	0.035	1.382	1.023	1.867
Chronic obstructive pulmonary disease	0.153	0.138	1.243	0.265	1.166	0.89	1.527
Malignant tumor	0.154	0.112	1.893	0.169	1.166	0.937	1.452
PP_24h_ > 70 mmHg	0.504	0.134	14.173	< 0.001	1.656	1.274	2.153
PP_24h_ < 40 mmHg	0.39	0.113	11.785	0.001	1.476	1.182	1.844
White blood cell count ( × 109/L)	0.005	0.004	2.012	0.156	1.006	0.998	1.013
Hemoglobin (g/dL)	0.099	0.017	34.012	< 0.001	1.104	1.068	1.141
Blood platelet (×109/L)	0.001	0	7.025	0.008	1.001	1	1.002
Alanine aminotransferase (U/L)	0	0	1.024	0.312	1	1	1
Creatinine (mg/dL)	0.042	0.026	2.497	0.114	1.043	0.99	1.098
Serum potassium (mmol/L)	–0.083	0.049	2.855	0.091	0.92	0.835	1.013
Serum sodium (mmol/L)	–0.023	0.007	10.211	0.001	0.977	0.963	0.991
SOFA	0.079	0.015	27.189	< 0.001	1.082	1.051	1.115
SAPS II	0.028	0.004	60.735	< 0.001	1.028	1.021	1.035
lactic acid (mmol/L)	0.107	0.016	47.094	< 0.001	1.113	1.08	1.148

## 4 Discussion

The Sepsis-3.0 guidelines particularly emphasize the central role of MAP in blood pressure management (2). However, in clinical practice, we observe significant individual variation in patients’ responses to treatment, even when MAP is elevated through fluids and vasoactive agents during the early resuscitation phase (14). Specifically, this variation may manifest as an increase in systolic blood pressure alone, an increase in diastolic blood pressure alone, or an increase in both simultaneously (15). These distinct patterns of blood pressure response indicate that achieving target MAP values alone is insufficient for effective blood pressure management; a more nuanced analysis is required. Studies indicate peripheral PP correlates with stroke volume and arterial stiffness (16). Extensive research on pulse pressure now demonstrates that both excessively high and low PP increase the incidence of major cardiovascular and cerebrovascular events, correlating with poor outcomes (17–19). Concurrently, Lee et al. examined the relationship between pulse pressure and 28-day mortality during initial resuscitation in 340 patients with septic shock, finding pulse pressure to be independently associated with 28-day mortality (20). Another study identified an increased 30-day mortality risk in patients with septic shock presenting pre-hospital pulse pressure below 40 mmHg. Researchers observed that employing PP as a non-invasive surrogate marker for cardiac output facilitates early optimization of cardiac output and tissue perfusion (21). Consequently, PP may to some extent reflect the circulatory status of septic shock patients, thereby aiding in the refinement of resuscitation strategies. However, definitive research on the optimal pulse pressure range during septic shock resuscitation remains lacking. This study, based on the MIMIC database, conducted an in-depth analysis of data from 3,668 patients with septic shock. The research focused on the impact of pulse pressure on both long-term and short-term mortality rates and survival curves, while accounting for potential confounding factors such as MAP, age, white blood cell count, and hemoglobin levels. Analysis revealed that patients with a pulse pressure range of 40–70 mmHg exhibited the most favorable prognosis. This may be attributed to inflammatory mediators and endotoxins damaging vascular endothelial cells, inducing the release of nitric oxide and endothelin, thereby causing microvascular smooth muscle dysfunction and vasodilation, which subsequently affects pulse pressure level (22, 23). During mild inflammatory responses, adequate fluid resuscitation typically yields hemodynamics characteristics of normal or increased cardiac output with reduced peripheral resistance, manifesting as elevated PP. Inadequate treatment or progression to septic shock induces excessive inflammation, leading to severe hypovolemia, myocardial suppression, and microthrombosis from coagulation abnormalities, exacerbating organ dysfunction (24, 25). These complications further diminish stroke volume, ultimately manifesting as extremely low PP. Thus, elevated PP may indicate controlled infection and favorable prognosis, though excessively high PP may correlate with unnecessary medication use. Research indicates that excessively high PP increases vascular wall tension, causing fatigue and rupture of elastic components, which further impairs vasomotor function and induces vascular remodeling (26). Moreover, the shear forces generated by excessive PP can induce endothelial cell damage. Inflammatory mediators released from damaged endothelium may indirectly impair microcirculation and exacerbate organ dysfunction (27). Thus, both excessively high and low pulse pressures may indicate an unfavorable prognosis. Therefore, PP may not only reflect the severity of septic shock but also serve as a complementary indicator for assessing treatment efficacy. However, it should be noted that this study did not account for vasopressor exposure, which directly influences pulse pressure. Therefore, during the recovery phase of septic shock, alongside maintaining MAP above 65 mmHg, pulse pressure within the range of 40–70 mmHg may be considered as a complementary reference, but further studies are needed to validate this finding. This facilitates patient recovery, a conclusion preliminarily validated by local databases. Previous studies indicate that age, gender, history of hypertension, history of myocardial infarction, and history of diabetes mellitus are key factors influencing pulse pressure, affecting it through multiple mechanisms. For instance, with advancing age, vascular elasticity diminishes, leading to elevated systolic pressure and reduced diastolic pressure, thereby increasing the pulse pressure difference. Gender differences also significantly influence the presentation and outcomes of cardiovascular diseases, potentially linked to variations in sex hormone levels, fat distribution, and metabolism. Hypertensive patients experience increased arterial stiffness due to atherosclerosis, which further affects pulse pressure. Myocardial infarction may impair cardiac pumping function and alter pulse pressure (28, 29). Diabetic patients frequently exhibit vascular endothelial dysfunction, heightened inflammatory responses, and atherosclerosis, all of which influence pulse pressure levels and the prognosis of sepsis (30, 31). Given these factors’ substantial influence on pulse pressure, this study further investigated whether pulse pressure consistently impacts prognosis in septic shock patients under specific conditions, such as differing genders or age groups. To this end, subgroup analyses were conducted across three distinct pulse pressure groups, revealing a significant association between pulse pressure levels and clinical outcomes in septic shock patients. Age emerged as a key factor influencing this relationship. Compared to PP < 40 mmHg, maintaining excessively high pulse pressure (PP > 70 mmHg) in elderly patients aged > 65 years was associated with poorer outcomes, whereas the opposite was true in younger patients. This discrepancy may relate to differing physiological characteristics between age groups. Elderly patients often exhibit reduced vascular elasticity and diminished cardiac function; excessively high pulse pressure may increase afterload, exacerbating cardiac burden (32). Conversely, younger patients possess greater vascular elasticity and cardiac compensatory capacity. Higher pulse pressure may thus better sustain tissue perfusion and oxygen delivery, thereby improving prognosis. This finding suggests that hemodynamics management in septic shock patients should incorporate age-specific adjustments to achieve optimal therapeutic outcomes. Several limitations must be acknowledged. As an observational study, we report associations, not causation. It remains unknown if actively targeting this PP range improves outcomes. Pairwise PSM allows for isolating specific associations but prevents direct statistical comparison between the High and Low PP groups against each other. Blood pressure data combined invasive and non-invasive methods. While reflective of real-world practice, non-invasive cuffs may be less accurate at hemodynamic extremes. Since PP is derived from SBP and DBP (which determine MAP), and MAP was a covariate, some mathematical coupling is unavoidable. Factors such as specific dosing of inotropes or fluid responsiveness status were not fully captured. The study included multiple exploratory analyses (e.g., 3-day/90-day mortality, subgroup analyses) without adjustment for multiple comparisons, which may increase the risk of Type I error. These exploratory findings should be interpreted cautiously and require further validation in future studies. The study did not incorporate detailed data on vasopressor exposure (including type, dose, intensity, and duration) into the analyses. This is a significant limitation because vasopressors directly influence systolic and diastolic blood pressure and thus pulse pressure. Differences in vasopressor requirements may reflect underlying shock severity, treatment intensity, or pharmacologic effects rather than intrinsic hemodynamic phenotype. Without accounting for vasopressor exposure, it is difficult to determine whether pulse pressure independently reflects prognosis or serves as a surrogate for treatment intensity or disease severity. This limitation has direct implications for the interpretation of the reported associations.

## 5 Conclusion

In summary, this study demonstrates that maintaining a mean PP within the reference range of 40–70 mmHg during the first 24 h of septic shock is consistently associated with improved survival outcomes. Both narrowed (<40 mmHg) and widened ( > 70 mmHg) pulse pressures serve as robust, independent indicators of poor prognosis. These findings suggest that Pulse Pressure may be considered alongside MAP as a complementary, widely available marker for hemodynamic risk stratification, particularly when interpreting hemodynamic status in elderly populations. However, the limitations of this study, including the lack of adjustment for vasopressor exposure and the exploratory nature of some analyses, should be taken into account when interpreting these results, particularly when interpreting hemodynamic status in elderly populations.

## Data Availability

Publicly available datasets were analyzed in this study. This data can be found at: This study utilized publicly available datasets, which can be accessed at https://mimic.mit.edu/. The internal database used in this study is available from the corresponding author upon reasonable request.
